# Supplementary material for: Are carbon emissions trading and green financial instruments synergistic? -Comprehensive quantitative research based on content analysis
Source: PLoS One. 2024 Mar 7;19(3):e0298601. doi: 10.1371/journal.pone.0298601 (PMC10919601; doi:10.1371/journal.pone.0298601)
Supplement: S1 Data — (ZIP) [file pone.0298601.s001.zip › Data/地级市Z_F工作报告.zip!/║╙▒▒/▓╫╓▌╩╨/└┤╫╘╣1⁄2╓┌║┼í╛┬φ┐╦╩2╛▌═°í┐_2010.docx]

各位代表：

我代表沧州市人民政府，向大会作政府工作报告，请予审议，并请各位政协委员和其他列席会议的同志提出意见。

一、2009年工作回顾

刚刚过去的一年，是新世纪以来我市经济发展最为困难的一年。在省委、省政府和市委的正确领导下，我们坚持以科学发展观为指导，紧紧围绕保增长、调结构、上项目、惠民生、促和谐，团结带领全市人民，万众一心，奋力拼搏，努力克服金融危机带来的不利影响，全市经济社会实现平稳较快发展，较好地完成了十二届人大二次会议确定的各项任务目标。全年地区生产总值预计完成1900亿元，增长11.5%，高于全省1.5个百分点。全部财政收入首次突破200亿元大关，达到210.3亿元，增长33.7%，跨入全省前三名。一般预算收入64.7亿元，增长14.1%。城市居民人均可支配收入14518元，增长10.2%，农民人均纯收入5000元，增长10%。

——港、产、城同步推进，渤海新区开发建设实现新跨越。去年3月19日，全市瞩目的黄骅综合大港正式开工，完成投资60亿元。围堰、防波堤、南北防沙堤等公用基础设施全部建成，码头主体工程初具规模，航道建设顺利推进，投资强度和建设速度创全国港口建设新纪录。今年8月1日，综合大港将如期开航，沧州人民多年的大港梦正一步步变成现实。集疏运配套体系建设加快推进，南疏港路、中疏港路建成通车，沧津沿海高速公路开工建设，邯黄铁路即将开工，保港、邯港高速公路前期工作进展顺利。产业聚集明显提速，中铁600万吨装备新材料、中钢8万吨镍铁、达力普100万吨铸锻等项目开工建设；沧东电厂二期2×66万千瓦机组、沧州大化5万吨TDI、金牛化工8万吨离子膜烧碱等项目竣工投产；渤海2×30万千瓦热电、正元化工60万吨醇氨、沧州大化10万吨TDI等项目前期工作扎实推进。16平方公里的黄骅生态新城起步区建设正式启动，实施以“5+5”为主的路网建设工程，总投资26亿元的“两纵三横”城市道路实现当年开工建设，当年竣工通车。

——重点项目建设步伐加快，投资规模实现新突破。全社会固定资产投资首次突破1000亿元，达到1102亿元，增长39.1%。“三五八十”在建和竣工项目210个，总投资1892亿元。京沪高铁、沧京、大广、沧津沿海高速公路等重大基础设施项目进展顺利。中原管道和中孟管件制造基地等117个产业支撑项目开工建设。沧海高压管件、中石油青县装备公司大口径埋弧焊管等54个大项目竣工投产。尤其振奋人心的是，一批改写沧州历史的大项目相继建成或开工建设：黄骅综合大港的开工建设，改写了沧州没有综合大港的历史；华北石化千万吨炼油项目开工建设，改写了沧州没有大炼油的历史；中铁、中钢两大项目的落户，改写了沧州没有现代大型钢铁企业的历史；杨埕水库建成蓄水，改写了沧州东部地区长期缺水和人民群众饮用苦咸水的历史。

——三年大变样活动成效显著，城乡建设呈现新面貌。坚持大手笔规划、大规模投入、大力度推进，进行了有史以来最大规模的城市改造和建设。市、县两级城市总体规划修编工作基本完成。拆迁拆违1017万平方米，提前一年完成省下达的任务。总投资142亿元的市区十大城建工程进展顺利。沧州体育馆、会展中心、狮城公园、金狮大酒店等一批标志性建筑投入使用，填补了市区没有大型体育馆、展览馆、休闲广场和五星级酒店的空白，拉开了沧州新城建设的序幕，也激发了全市人民热爱家乡、建设沧州的极大热情。新建、改造道路10条11.4公里，改造小街小巷40个。铺设供水、燃气、供热管网68公里。新增供热面积80万平方米，总面积达到900万平方米，集中供热率67%，市区空气质量明显提高，二级以上天数达到330天，同比增加9天。小南门、颐和广场两大商业中心加快建设，13个标准化菜市场建成投用。新增星级宾馆10家。启动改造城中村15个。颐和文园、阿尔卡迪亚等住宅小区加快推进。市区商品房竣工面积53.3万平方米，经济适用房13.1万平方米，广厦家园等1942套廉租住房建成入住。新增和改建绿化面积164万平方米，建成区绿化覆盖率达到38%，跻身全省先进行列。解放路景观改造按时完成。手机3G业务正式开通。总投资82亿元的县城“十个一”工程稳步推进，谋划实施项目96个，完成投资49亿元，县城功能不断完善，县城面貌明显改观。新民居建设步伐加快，河间李庄、青县耿官屯、献县小屯等37个新建村主体工程基本竣工。坚持一手抓城市建设，一手抓城市经济，在加快推进沧州开发区东扩、沧东工业园建设及高新技术开发区启动的同时，大力发展中心城市服务业。东来顺、全聚德等名品名店落户沧州，沧州综合物流园、彩龙国际商贸城前期工作扎实推进。中心城区第三产业增加值完成170亿元，增长16.1%，同比提高1.6个百分点；占市区生产总值的49.3%，比上年提高3个百分点，为壮大中心城市经济和市本级财力奠定了基础。

——“一节一会”成功举办，对内对外开放增添新活力。坚持“文化搭台、经贸唱戏”，建设一流的场馆设施，开展一流的组织服务，打造一流的平安环境。成功举办了第12届中国吴桥国际杂技艺术节沧州会场和中国·沧州管道装备展览会，搭建了对外开放的新平台，彰显了“石油之城、管道之都”的风采，扩大了沧州的知名度、美誉度，同时催生了会展经济，提振了全市人民的精神和信心。展会期间，与中石油、华北油田、大港油田等6家企业签订了战略合作协议，与榆林市签订了政府间合作协议，举办了管道装备制造业发展高层论坛。签署合作项目40个，总投资164亿元，取得交易和合作成果70亿元。积极开展招商引资活动，全年直接利用外资1.9亿美元；引进省外资金128亿元。基地和园区建设取得新成效，沧州开发区、任丘开发区、化工园区完成扩区审批，泊头、青县、盐山、孟村、献县等11个工业园区被批准为省级产业聚集区，全市重点园区入区企业达到3018家。

——工业经济企稳向好，县域经济得到新提升。深入开展“访企问计”送温暖活动，加大对重点企业的指导和生产要素的协调调度，工业经济实现了逐月回升和平稳增长。全市规模以上工业企业完成增加值585亿元，增长11%。其中，“三五八十”重点企业完成410亿元，占全市规模以上工业增加值的70%，增长11%。规模以上工业企业完成利税280亿元，增长24%，比全省增速高26.4个百分点，居全省第一位。企业自主创新能力不断增强，河北华斯、新宝丰线缆等4家研发中心被列入省级重点研发中心。任丘京东集团“京东”牌商标、盐山中原集团“世兴”牌商标被评为中国驰名商标。县域经济发展势头强劲，“三五八十”大项目、大企业明显增多，产业集聚步伐加快，特色更加突出，全年实现地区生产总值1388亿元，占全市经济总量的73%。

——农村经济持续发展，产业化经营迈出新步伐。全市粮食总产451万吨，增长4.6%，连续6年增产增收。畜牧、蔬菜两大产业快速发展。出栏肉鸡4200万只，增长33%；发展节水灌溉面积33万亩，新增设施蔬菜种植面积3万亩，新增造林面积30万亩，建设果品创汇基地55万亩。产业化经营步伐明显加快，农民专业合作组织达到1121个。任丘大发、孟村大成等产业化项目顺利推进，完成投资32亿元，省级以上农业产业化龙头企业达到33家，产业化经营率达到50%。认真落实各项惠农政策，发放粮食和农资直补及良种、农机具、燃油等各类补贴9.9亿元。新修、改建农村公路1048公里，新建、维修道路桥梁50座，改造危险闸桥158座。生态家园富民工程扎实推进，新建各类沼气池3.2万个。“万村千乡”市场工程新建、改造农家店1157家，农村流通网络日趋健全。

——金融业发展迅速，投融资平台建设开创新局面。全市金融机构存贷款余额分别达到1765亿元和897亿元，累放贷款1071亿元，新增贷款规模325亿元，同比增加3倍，存贷差逐年缩小，贷款存量结构明显改善。沧州银行总资产达到157亿元，成为全省首家晋升二级行的地方商业银行。民生银行、交通银行落户沧州，河北恒银期货沧州营业部正式开业，填补了我市金融期货市场的空白。沧州建设投资公司总资产达到120亿元，渤海投资公司超过100亿元。市建投担保公司正式成立，18个县（市、区）全部建立了投资公司和担保公司。小额贷款公司快速发展。会友电缆在天交所成功上市。沧州被省政府授予“金融生态市”称号。

——节能减排成效明显，可持续发展能力得到新提高。强力推进“十大节能”工程，100家重点企业节能降耗取得积极成果，省“双三十”和重点领域、重点企业的监管力度不断加大，“8755”减排工程和环保“利剑”行动成效明显。除渤海新区环境容量指标尚未经省政府核定外，预计全市万元GDP能耗下降5.2%。化学需氧量、二氧化硫排放量分别削减1.47万吨和0.82万吨。各县（市、区）污水处理厂和垃圾处理场全部开工建设，8个污水处理厂建成投用。耕地保有量和基本农田面积得到有效控制，土地监管利用水平不断提高。

——民生工程扎实推进，各项社会事业取得新成绩。全面落实以县为主的义务教育经费保障机制，义务教育阶段学校教师绩效工资基本落实。大力实施校舍安全工程，创建标准化小学133所，建成布局调整项目学校20所，新建、改扩建校舍19.2万平方米。沧州师专成功升本，结束了我市没有本科院校的历史。二类城市语言文字达标工作高质量通过省级验收。50个乡镇综合文化站、309个农村书屋全部建成。实施扶贫开发项目486个，落实扶贫开发资金2.1亿元。“六险”覆盖面稳步扩大。城镇居民基本医疗保险参保人数40.4万人。住房公积金累计归集53亿元，缴存覆盖率达到87.5%。新型农村合作医疗参合农民512.4万人，参合率达到93%，为参合农民报销医药费4.9亿元。为17.4万城乡低保对象发放低保金2.1亿元。全市90所敬老院、光荣院、福利院全部建成，并达到三星级入住标准，集中供养能力达到90％，进入全省、全国先进行列。城镇新增就业4.6万人，城市登记失业率控制在4.5%以内。甲型H1N1流感防控救治工作取得阶段性胜利。农村改厕“百村万户”工程扎实推进，建造卫生户厕2.2万个。“计生惠民”行动深入实施，人口自然增长率控制在7‰以内。民族宗教工作成绩突出，我市被国务院授予民族团结进步模范集体称号。大力开展安全生产和食品药品质量安全专项整治，安全生产形势总体良好，食品药品质量安全进一步改善。深入开展社会治安综合治理，加大对各类违法犯罪活动的打击力度，认真做好“国庆”和“一节一会”期间的安全保卫工作，安定和谐的社会局面进一步巩固。统计、人防、档案、气象、地震、海关、海事、港航、检验检疫、外事旅游、广播电视等各项事业都取得长足进步。

——依法行政水平不断提高，政府自身建设取得新成效。深入开展学习实践科学发展观暨干部作风建设年活动，干部作风进一步好转。市级政府机构改革基本完成。行政审批制度改革不断深化，削减市本级行政许可项目109项。继续加强机构编制管理，严格控制财政供养人员数量。深入推进政务公开和行政权力公开透明运行。加大审计、监察工作力度，严肃查处了一批违法违纪典型案件。主动向市人大、市政协报告工作、通报情况，共办理市人大代表建议98件，市政协委员提案271件，办复率100%。

各位代表！过去一年，是我们积极主动应对国际金融危机，在危机中抢抓机遇、在困境中寻求突破、在竞争中赢得发展的一年；是我们打造大港口、发展大交通、修建大水库、建设大城市，推进沿海强市建设，提升沧州战略地位的一年；是我们发展大炼油、培育大钢铁，提升产业层次和综合竞争实力的一年；是我们体察民情、顺应民意、改善民生，大力推进和谐沧州建设的一年。一年来，许多在过去看来难以克服的困难被我们克服了，许多过去我们想都不敢想的事情办成了。实践充分证明，只要我们坚定不移地贯彻落实科学发展观，坚定不移地贯彻落实省委、省政府和市委的决策部署，坚定不移地从沧州实际出发，咬定发展不放松，抓住项目不放手，全市上下团结一心，凝神聚力，心无旁骛，拼搏进取，真抓实干，一刻也不懈怠，一天也不耽误，我们就一定能够不断开创沧州经济社会发展的新局面！

各位代表！过去一年成绩的取得，是中共沧州市委正确领导的结果，是市人大、市政协鼎力支持的结果，是全市各级干部共同努力奋斗的结果，是全市人民团结一心、攻坚克难的结果。在此，我代表沧州市人民政府，向辛勤工作在各条战线上的全市各族人民，向给予我们大力支持的人大代表、政协委员和各民主党派、工商联、无党派人士，向大力支持我们工作的离退休老干部、驻沧各单位、人民解放军和武警官兵，向所有关心、支持沧州发展的同志们、朋友们，致以崇高的敬意和衷心的感谢！

回顾过去一年的工作，我们也清醒地看到，发展进程中还存在一些不容忽视的矛盾和问题。一方面，全市上下各级干部解放思想的步子还不够快，改革开放的力度还不够大，发展是第一要务的理念树得还不够牢，各级各部门服务发展、服务大局的作风还不够扎实，发展市场经济的办法和科学发展的能力和素质与沧州快速发展的形势还不适应，吃拿卡要和阻工现象时有发生，投资环境有待进一步优化和改善。另一方面，全市经济发展中“两小一低”问题比较突出，经济总量小，企业单体规模小，产业层次低。财政收入结构性矛盾突出，特别是市本级经济权重小、财力弱。产业结构调整、节能减排任务艰巨。对这些矛盾和问题，我们将高度重视，认真加以克服和解决。

二、2010年总体要求和发展目标

目前，沧州已经进入新的历史发展阶段。站在新的历史起点，我们必须强化新理念，开拓新思路，明确新目标，寻求新突破，实现新跨越。今后一个时期，我们的发展方向和战略目标是：抢抓机遇，乘势而上，努力把沧州打造成为冀中南区域发展的增长极和隆起带。

纵观国内外发展大势和我市实际，我们面临着十分难得的发展机遇。从国际看，国际金融市场渐趋稳定，世界经济有望实现恢复性增长。从国内看，我国经济回升向好的基础逐步巩固，中央继续实施积极的财政政策和适度宽松的货币政策，保持宏观经济政策的连续性、稳定性，扩大内需和改善民生的政策效应将进一步显现。从全省看，省委、省政府高度重视沿海经济和“一线三区”的发展，把沧州渤海新区作为河北新的经济增长极，并给予大力支持。从沧州自身看，我们已经具备了科学发展、率先发展、跨越发展的基础和条件，进入了厚积薄发的历史新时期：第一，随着黄骅综合大港的开航，我市将成为冀中南地区名副其实的“东出西联”出海口。黄骅综合大港的建成通航，使沧州真正成为“东出西联”的桥头堡。特别是邯黄铁路的开工建设，保港高速、邯黄高速的快速推进，沧州可在更大范围内整合资源，聚集生产要素，加快建设石油化工和现代装备制造产业聚集区，从而奠定在冀中南区域发展中的龙头地位。第二，随着京沪高铁、沧京高速的竣工通车，我市将真正融入京津一小时经济圈。京沪高铁将于今年试运行，届时从沧州到北京只需50分钟，到天津只需30分钟。沧京高速也将于年底竣工。独特的交通优势将进一步转化为经济优势、发展优势，沧州可以更快速、更便捷地利用京津的资金、技术、人才和信息资源，从而进入与京津“无缝对接”的新时代。第三，随着大炼油、大钢铁、大装备等一批重大产业支撑项目相继开工和建成投产，为我市经济发展提供了强有力的支撑。我市已拥有“三五八十”大企业、大集团115家，销售收入超100亿元的4家。以石油化工、装备制造和冶金为主的现代产业体系初步形成，特别是华北石化、中石油青县装备、鑫源泰、中铁、中钢等一批产业支撑项目的加快建设，将推动三大产业迅速提档升级、膨胀发展，成为沧州经济发展的强劲支撑。第四，随着多年的积累和发展，我市已经具备了打造冀中南区域发展增长极、隆起带的实力和能力。我市经济总量已连续四年位居全省第四，年增长速度均保持两位数。特别是今年我市财政收入突破200亿元大关，位列全省第三，我市经济已经进入快速发展的新阶段，沧州将备受世人关注，知名度、影响力将大大提高。同时，各级领导干部在应对金融危机、开展三年大变样活动、举办“一节一会”和重点项目建设中，得到了锻炼，积累了经验，推动科学发展和驾驭复杂局面的能力明显提高，为打造冀中南区域发展的增长极、隆起带奠定了坚实基础。

总之，我们一定要增强机遇意识，坚定发展信心，努力在新一轮发展中，把握新优势，抢占制高点，打造冀中南区域发展增长极和隆起带，实现科学发展、率先发展、跨越发展。这既是省委、省政府的希望和重托，又是710万沧州人民的厚望和期盼，更是我们义不容辞的政治责任和光荣使命。我们必须要有这样的雄心壮志，我们必须担当起这一历史重任，用我们的不懈努力和无私奉献来创造沧州发展的新辉煌！

2010年，是“十一五”规划的最后一年。做好今年的各项工作，对保持经济平稳较快发展，为实施“十二五”规划奠定良好基础，具有十分重要的意义。今年，政府工作的总体要求是：全面贯彻党的十七大、十七届四中全会精神，高举中国特色社会主义伟大旗帜，以邓小平理论和“三个代表”重要思想为指导，深入贯彻落实科学发展观，以保增长、保投资为主线，更加注重解放思想、改革开放，更加注重自主创新，提高综合竞争力，更加注重转变发展方式和构建现代产业体系，更加注重节能减排和环境保护，更加注重统筹城乡区域发展，更加注重社会保障和改善民生，加快工业化、城市化和农业产业化步伐，突出渤海新区龙头带动、城市化建设突破提升、县域经济腾飞升位三项重点工作，推动全市经济社会平稳较快发展，努力把沧州打造成为冀中南区域发展的增长极和隆起带。

主要预期目标是：地区生产总值增长11.5%；全社会固定资产投资增长30%以上；全部财政收入增长13%，地方一般预算收入增长15%；城市居民人均可支配收入增长11%，农民人均纯收入增长9%。

实现上述目标，一个重要前提就是解放思想。思想解放的程度决定经济发展的速度，没有思想的大解放，就没有经济的大发展、社会的大进步。我们必须打开解放思想的总开关，扭住转变观念的总阀门，敢想、敢干、敢闯、敢试。敢为人先、敢于担当、敢作敢为，想别人不敢想的事、想不到的事，干前人没干过的事、干不了的事。在解放思想的基础上，努力做到“两保”、“两转”、“三个提升”和“三个更加关注”。

两保：一是保增长。打造增长极、隆起带，需要保增长；解决发展中的矛盾和问题，需要保增长。作为正处于速度效益型、投资拉动型发展阶段的沧州来讲，不增长就没有出路，不增长高质量发展就无从谈起。要始终坚持把保增长作为今年政府工作的首要任务，既要积极做大增量，又要努力盘活存量，加快培育新的经济增长点，确保发展的态势不变、速度不减，主要经济指标再上新台阶。二是保投资。保投资是保增长的重要手段。没有投入就没有发展，没有投入就没有希望，没有投入就没有沧州的未来，这是抓发展的重中之重。我们必须扭住投资这个牛鼻子，推进重点项目建设不动摇，推进“三五八十”工程不放松，确保更大的投资规模和更快的增长速度。

两转：一要转变发展方式。发展方式决定发展内涵和质量，决定一个地区的综合竞争力。我们必须把加快经济发展方式转变，作为深入贯彻落实科学发展观的重要目标和战略举措来抓，坚持好字当头，又好又快，科学发展，加快由高耗能、高污染、低效、低附加值向低能耗、低污染、高效、高附加值转变，由粗放型向集约、集成发展转变，由劳动密集型向资金、技术密集型发展转变。二要转变发展模式。我们不能长期停留在过去乡镇企业发展的阶段和水平上，必须打破传统的发展模式，积极引进战略投资者，通过引进项目和资本运作，推进战略重组，由加工型、依附型、配套型向制造型、终端产品型、资本型转变，由配角向主角转变，由集体打工向企业老板转变，实现跨越式发展。

三个提升：第一，提升产业层次。产业链条短、产品低端低档、配套能力不强，是我市产业发展面临的突出问题。必须以构建现代产业体系为目标，上规模、上装备、上管理、上水平，逐步实现产业链条向高端升级，产业布局向集群发展，工艺装备向现代化迈进。第二，提升企业规模。企业是经济发展的重要支撑。必须坚持一手抓顶天立地，一手抓铺天盖地；一手抓增量，一手抓存量；一手抓引进，一手抓再造，加快企业升位，培育一批支撑沧州发展、支撑沧州财政的大企业、大集团，打造企业“航母”。第三，提升园区水平。园区是对外开放和产业聚集的平台，但这始终是我市经济发展的一个“短板”，必须尽快“补课”，力求赶超。进一步配强园区领导班子，在管理体制上实现突破；加快基础设施建设，在完善功能上实现突破；完善规划，明确产业定位，引进一批“三五八十”战略支撑项目，在产业聚集上实现突破。

三个更加关注：更加关注“三农”。农业是国民经济的基础，任何时候都不能放松农业和农村工作，必须以发展工业的理念发展现代农业，以城镇化的思维推进社会主义新农村建设，加快农业产业化步伐，不断提高农民的组织化程度，促进农业增效、农民增收、农村稳定。更加关注社会发展。坚持协调发展、和谐发展，进一步加大投入，积极发展教育、卫生、文化、体育等各项社会事业。强化公共和基层管理，夯实社会发展基础，推进公安“三基”及农村和社区基层组织建设，在办公条件、经费开支、人员配备等方面提供有力保障。更加关注民生。发展经济的目的是保障和改善民生，我们必须坚持以人为本，把解决好民生问题作为最大的政治、最大的政绩，完善政策措施，加大投入力度，着力解决就业、保障、住房等事关人民群众切身利益的问题，让全市人民充分享受改革发展的成果。

今年经济社会发展的任务十分繁重，必须把握全局，统筹兼顾，抓住关键，重点突破。着力抓好以下十个方面的工作：

（一）扬渤海新区龙头，大力发展沿海经济。强化渤海新区龙头地位，率先解放思想、率先科学发展、率先创新体制机制，切实发挥龙头带动示范作用、创新发展方式作用、创新体制机制作用、创新发展环境作用，当好全市经济社会发展的排头兵。按照渤海新区三个发展阶段的战略构想，努力将渤海新区由省级层面上升为国家层面。推进港口、港区、港城、腹地统筹协调发展，全力打好“十大战役”。一是加快综合大港建设。以8月1日综合大港开航为目标，加快码头、航道建设，并做好开航和大通关前的各项准备工作。推进集装箱码头、矿石专业码头、油码头、化学品码头建设，为实现综合大港“三步走”战略目标奠定基础。加快邯黄铁路、沧津沿海高速公路建设，积极谋划保港和邯黄高速公路，构建以综合大港为龙头的集疏运体系。二是加快临港产业发展。积极探索“一区多园”的管理模式，进一步搞好产业发展规划，谋划建设一批产业支撑项目。加快中铁、中钢、达力普铸锻等项目建设，确保早日竣工投产；全力推进中捷石化1000万吨炼油、沧东电厂三期、石钢搬迁等项目前期工作；开工建设渤海2×30万千瓦热电、金牛化工40万吨PVC、沧州大化10万吨TDI等重点项目。三是加快黄骅生态新城建设。按照高起点规划、高质量建设、高水平管理的要求，抓紧编制各项控制性详规及专项规划，加快新城水、电、路、气、讯等基础设施建设，推进总投资90亿元的明珠大桥、港城快速路等十大起步工程。四是加快港区服务设施建设。推进航运中心大厦、渤海港务公司调度楼、11万伏变电站、供水管线，以及学校、医院、商业、文化、娱乐等配套设施建设，保证综合大港运营需要。五是加快城中村改造步伐。坚持统一规划、整体拆迁，推进组团式开发，力争3年新区城镇化率达到100%。六是加快港口腹地开拓。委托高水平的研究咨询机构开展黄骅港经济腹地发展研究，深化与腹地城市和各大企业间的合作，积极组织好渤海新区高层论坛。七是加快保税港区建设。尽快启动渤海新区保税区建设，抓紧B型保税物流中心的申报，推进港口物流和国际贸易发展。八是加快南大港旅游区建设。编制旅游区发展规划，以南大港湿地为依托，搞好景点开发和旅游配套服务设施建设。九是加快中心商务区建设。按照适度超前原则，加快企业总部、金融总部建设。十是加快高教园区建设。重点推进北京交通大学海滨学院、北京医药大学东方学院、中国地质大学长城学院、河北农业大学渤海校区建设。

（二）强化投资拉动，毫不动摇地抓好重点项目建设。深入实施“三五八十”工程，全力推进一批重大基础设施和产业支撑项目。一是加强重点项目谋划储备。围绕工业倍增计划，谋划好100万吨乙烯、100万吨芳烃、100万吨甲醇、20万吨MDI、中原大口径管道、沧东电厂三期2×100万千瓦机组、海兴核电站等一批产业支撑项目；围绕提升区域综合竞争力，谋划好沧州至石家庄、渤海新区至滨海新区城际铁路，任德、邯港、保港高速公路、沧州支线机场等一批基础设施项目，争取更多项目列入国家和省“十二五”发展规划。二是加快重点项目前期跑办。着力推进中捷石化1000万吨炼油、正元集团100万吨二甲醚、浅海石油化工等项目前期工作，确保100个重大产业支撑项目开工建设。加快华润热电三号机组、渤海热电等前期项目的跑办，确保一批重大基础设施项目开工建设。三是扎实推进在建重点项目。加快华北石化1000万吨和沧炼500万吨炼油升级改造、东化100万吨尿素、渤海重工10万吨高压合金管、中原管道装备制造基地、宏润海水淡化设备、任丘热电等一批重大产业支撑项目建设，确保42个项目建成投产。加快京沪高铁、沧京高速、大广高速、沧津沿海高速等一批重大基础设施项目建设，确保10个项目竣工投运。四是优化重点项目建设环境。坚持节约集约原则，按照保发展、保红线的要求，有效增加项目供地。深入开展“两跑四争”活动。推进重点项目建设领导分包责任制和代理跑办制，加强重点项目警务室建设，推行“一卡通”挂牌保护制度，确保重点项目建设顺利推进。

（三）加快产业结构调整，积极构建现代产业体系。围绕“三个三、十个一”产业发展目标，深入实施“四大两升一换代”工程，着力构建现代产业体系，加快新型工业化步伐。一是加快产业升级，做大做强主导产业。1、石化产业：按照“油头化身”的思路，瞄准3000万吨炼油能力，推进华北石化1000万吨、中捷石化1000万吨、沧炼500万吨及南大港石化、鑫泉焦化等炼油质量升级改造。在做大“油头”的同时，努力延长“化身”，着力抓好1000万吨合成氨、100万吨PVC、100万吨二甲醚等十大系列化工项目，打造绿色、安全、国际石油化工城。2、装备制造业：管道装备制造业，瞄准3000万吨管道装备制造能力，以高压、高端、高附加值为目标，推动管道装备制造业由单一的管道、管件向石油化工设备、石油钻采装备和压力容器设备转变。重点推进中石油青县装备“神州第一管”、 鑫源泰X100管道、宏润风力发电和核电管道制造、达力普铸锻、渤海重工高压合金管等重大项目建设，打造国内外知名的管道装备之都。机械制造业，依托泊头机床、东光包装机械、海兴造船等产业基础，谋划建设一批新型机械制造项目，推动机械制造业由零件向部件、部件向整机转变。3、冶金产业：瞄准3000万吨装备新材料生产能力，抓住国家钢铁产业向沿海转移的有利时机，加快推进中铁、中钢和石钢搬迁等项目。积极创造条件，吸引更多冶金企业和项目向渤海新区聚集，打造冀中南装备新材料制造基地。二是推动企业升位，培育一批在全省乃至全国有影响的大企业、大集团、大品牌。结合我省实施的“对标行动”，鼓励企业采用新技术、引进新工艺、购置新设备，推动企业升位和产品更新换代。突出抓好总投资400亿元的70个技改项目，力争“三五八十”企业达到120家。积极推进重点企业建立产品检验检测中心和产品质量认证，扶持中原钢管、鑫源泰钢管、新宝丰线缆、沧州大化TDI等10家企业产品争创中国驰名商标和中国名牌。三是大力发展县域经济，培育壮大产业集群。坚持走集约集群发展道路，以大产业为骨架、大基地为依托、大园区为平台、大项目为载体，加快五金机电、汽车模具、食品加工、纺织服装等县域特色产业优化升级，推进项目向园区集中、园区向县城集中、人口向城市集中。落实好鼓励全民创业的政策和措施，努力营造促进全民创业和民营经济发展的良好环境，培育壮大市场主体，不断提升县域经济的综合实力。四是推进节能减排，促进发展方式转变。在抓好省“双三十”、“双百”企业的同时，深入实施“8755”减排和“十大节能”工程，继续抓好100家重点耗能企业的节能降耗工作。积极推进沧东电厂、化工园区、东光化肥等循环经济示范单位建设，探索并推行节能环保型发展模式。加快建设污水处理厂、垃圾处理场，确保年内全部建成运营。

（四）全力打好三年大变样攻坚战，推进城市化建设再上新台阶。以“两节”和“五城同创”为载体，以“双十”工程为抓手，围绕“一主两副多颗星”的城镇化发展格局，深入开展城镇面貌三年大变样活动，推进城市建设上规模、上档次、上水平。一是全面提高城乡规划水平。加大规划投入，进一步开放规划市场，实现城乡规划一体化、全覆盖。重点编制好中心城区新城规划和各类专项规划。加强规划队伍建设，完善规划审批管理机制，严格审批流程，强化规划监察，维护规划权威。二是全面提高城市建设水平。按照“四位一体、同步推进”的要求，加快推进中心城市、县城、小城镇和新民居建设。中心城市建设：围绕“一河、两岸、西文、中商、东工、满城绿”的发展思路和功能定位，加快实施总投资220亿元的中心城区十大城建工程，并加快推进沧县同城化工作。1、新城起步工程。开工建设“一场三馆”和“一中心两总部”。一场三馆，即体育场、博物馆、图书馆、武术馆；一中心两总部，即市民服务中心、金融总部和企业总部。2、道路畅通工程。新建京沪、石黄高速连接线等8条道路，拓宽改造新华路、解放路、交通大道部分路段，改造小街小巷35条。3、城市排水工程。启动代家园和光荣路泵站迁移及配套管网建设，完成浮阳北大道等7条排水渠清淤改造。4、住房建设工程。着力抓好御景狮城、天成瑞府等一批住宅小区建设。5、城中村及旧平房区改造工程。重点抓好大季屯、大和庄、王御史、蔡庄子等城中村，以及一中前街片区、火车站片区、荷花池片区、泰和世家等旧城区改造。6、绿化亮化美化及街道景观整治工程。开工建设名人植物园和迎宾大道西侧景观带，实施北京路、上海路等5条道路及高速公路出入口环岛绿化工程；抓好市区主要街道及高大建筑物的亮化；实施新华路、清池大道等街道景观整治。7、教育设施建设工程。新建、改扩建11所中小学，启动沧州一中迁建项目，开工建设河北工专新校区。8、民政设施建设工程。抓好现代、环保、绿色综合殡葬服务项目建设。9、集中供热及管网建设工程。全力推进华润热电3号机组开工建设，加快换热站及分支热力管网建设。新建供水、电力、燃气、通讯管线87.9公里。10、运河水系改造和景观整治工程。加快编制市区古运河开发、改造规划，推进运河水系改造和景观建设，启动运河两岸文物古迹修复工程。县城建设：重点抓好一所高级中学、一家二级甲等医院等“十个一”工程，进一步完善功能，提升品位。小城镇建设：重点推进泊头交河、河间米各庄等13个建制镇建设，进一步完善道路、照明、绿化、供排水等基础设施。新民居建设：重点抓好217个新民居示范村，并逐步向中心城市周边、县城周边、开发区和园区周边、国省干道两侧延伸。三是全面提高城市管理水平。按照精心、精细、精品的要求，从规划设计入手管理好城市。重点抓好环境卫生、“五乱”治理和景观改造，加快城市净化、绿化、亮化、美化、数字化步伐。进一步完善城市管理体制机制，权力下放，重心下移，发挥好两区、街道和社区的管理职能。深入实施市民素质提升工程，大力营造人民城市人民建、人民城市人民管、人民城市人民爱的浓厚氛围，建设宜居、宜商、宜业、宜学、宜游的新沧州，打响城市品牌。四是全面提高城市经济发展水平。坚持以产兴城、以城带产，在积极推进城市建设的同时，大力发展城市经济，壮大市本级可用财力。以打造石油钻采设备、石油化工装备制造基地为重点，加快沧州开发区、高新区和新华工业区、沧东工业园区建设，全力推进沧炼、达力普、鑫源泰、飞达、东塑等重点建设项目。以引进名品名店和大型物流集团为重点，加快发展商贸服务业，提升中心城市商贸服务业的龙头地位。

（五）积极发展现代服务业，着力扩大居民消费。坚持把服务业作为扩内需、保增长、调结构、增收入的重要环节，着力提高服务业发展水平和对经济增长的贡献率。一是大力发展生产性服务业。以中心城市为依托，加快发展金融保险、会展服务、教育培训、劳务输出等生产性服务业；围绕石油化工、装备制造、冶金等主导产业，积极谋划建设渤海新区国际综合物流园区；推进与河北物产集团等省内外大公司、大集团的战略合作，加快沧东、沧西等综合物流园区建设，打造以黄骅港为龙头的冀中南区域物流中心。二是大力发展生活性服务业。按照主城区商贸服务业发展规划，重点抓好京沪高铁新火车站商业广场启动建设和老火车站站前商业街、小南门等商业设施升级改造，加快颐和广场、一中前街商业广场、泰大和彩龙国际商贸城及8个标准化菜市场建设，构筑“一轴、两核、多层级、多业态”的新型商贸服务业体系。结合住宅小区建设，加快大型超市、社区连锁店建设，积极发展文化娱乐、家政服务、医疗保健、康复健身等新兴服务业。充分发挥杂技、武术旅游资源优势，搞好吴桥杂技大世界升级改造。加快白洋淀温泉度假村、滨海休闲观光等景点建设，打造精品景区和线路。三是大力发展农村商贸服务业。继续推进“万村千乡”市场工程，加快农家店和配送中心建设，完善农产品批发市场服务功能，积极鼓励农超对接。进一步做好家电、汽车摩托车下乡工作，落实国家直补政策，提高农村居民消费能力。

（六）着力做好“三农”工作，大力促进农业增效、农民增收。一是调整优化农业产业结构，做大做强畜牧、蔬菜产业。稳定粮食播种面积，确保总产450万吨以上。以畜牧、果品、蔬菜、棉花为重点，大力调整和优化农业结构，建设一批规模大、特色强、集约化程度高的种植、养殖基地。加大土地开发整理力度，划定永久基本农田，确保耕地保有量刚性目标。二是大力发展农民专业合作社，提高组织化程度。着力抓好100个市级示范社建设，新增农民专业合作社245家，力争全市三分之一的村建立农民专业合作社，三分之一的农户进入合作组织。三是扶优扶强龙头企业，提高产业化经营水平。全力推进肃宁顺鑫、沧县温氏、黄骅成润等10个超亿元农业产业化项目，突出抓好10家集团型龙头企业。发挥青县小洋人、泊头东方果品、河间国欣农研会等龙头企业带动作用，扩大优势农产品生产规模，延伸产业链条，提高专业化、标准化水平。四是加强农村基础设施建设，改善农业生产条件。大力推进农田水利基本建设，新增节水灌溉面积25万亩。搞好农业综合开发，力争开发项目投资比上年增加10%以上。深入实施“1536”造林绿化工程，新增植树造林面积20万亩。新建改建农村公路400公里、道路危桥13座，维修改建水利闸桥153座。推进农户科学储粮工程，完成农户小粮仓建设4.3万个。谋划建设9个沼气项目，新增农村沼气用户3.1万户，完成农村改厕1.9万个。五是落实惠农政策，努力增加农民收入。认真落实国家各项惠农政策，加大财政支农惠农力度。开展“一乡一业、一村一品、一人一技”活动，搞好农村劳动力转移输出，努力增加农民工资性收入。

（七）发挥“两环”优势，不断提高对内对外开放水平。我市既环京津又环渤海，打京津牌、吃沿海饭、念资本经、走产业路，是今后一个时期全市对内对外开放的总体要求，要进一步加大开放力度，加快构筑全方位、多层次、宽领域、内外融通的大开放格局。一是打京津牌，积极推进与京津的全面对接。抓住京沪高铁、沧京高速竣工通车，沧州进入京津一小时经济圈的重大机遇，精心谋划包装一批大项目，一对一，点对点，积极开展与国家部委、国内外500强企业、金融机构、科研院所和外国驻华商务机构的对接活动，推进与京津在基础设施、产业发展，以及资金、技术、人才、金融、旅游等多个领域全面合作。二是打沿海牌，加强与经济腹地的交流与合作。充分利用黄骅综合大港开航的有利时机，加大宣传推介力度，提高渤海新区和黄骅综合大港的知名度。坚持政府推动、企业参与，谋划并组织好港口与腹地特别是冀中南五市的合作推进会，在资金、技术、物流等方面开展全方位合作，互惠双赢，共同发展。三是打央企牌，积极引进战略投资者。巩固与“三油、五电、三锅”等央企的合作基础，进一步扩大合作领域和范围，围绕促进主导产业、特色产业发展，谋划一批产业接续项目、配套项目、转移项目，力争在与央企的战略合作、战略重组等方面取得更大突破。四是打园区牌，充分发挥基地园区对外开放的窗口和平台作用。着眼打造招商引资示范区、产业聚集示范区和自主创新示范区，加快十大基地、十大园区“九通一平”建设，完善功能，强化服务，增强对项目、资金等要素资源的吸纳能力。五是打节庆牌，全力办好中国·沧州（10·9）国际武术节暨中国·沧州（黄骅）冬枣节。搭武术台，唱经贸戏、开放戏、项目戏。在组织好武术赛事和冬枣采摘及旅游观光等活动的同时，举办沧州经济贸易洽谈会，通过项目洽谈对接活动，扩大合作，推动招商引资工作再上新台阶。

（八）深化重点领域改革，进一步完善体制机制。改革是促进发展的不竭动力，要毫不动摇地深化改革，向改革要动力，向改革要效益，向改革要发展。一是深化国有企业改革。加快国有工商企业破产终结和改制扫尾工作。进一步完善法人治理结构，强化国有资产监管，确保国有资产保值增值。二是深化公用事业改革。抓好城市园林、环卫、燃气、供热、供排水等公司制改革，吸纳社会资金，激活国有资产，变以钱养人为以钱养事。三是深化文化体制改革。创新公益性文化事业和经营性文化产业的体制机制，推进经营性文化单位的转企改制。整合杂技、武术资源，组建杂技、武术企业集团，打造、提升两大文化品牌。四是深化财税金融体制改革。优化财政收入结构，研究探索提高财政贡献率和市本级可用财力的可行办法。优化财政支出结构，加快构建公共财政体系，把新增财力更多地用于教育、卫生、社会保障等公共服务领域。整合资源，做大做强“一行两投”，探索组建市政工程集团公司、高速公路集团公司，提高投融资能力。积极发展小额贷款公司和股份制担保机构，加快组建农村商业银行。大力引进国内外银行、保险、证券等金融机构。力争建新化工等3家企业成功上市，三井酒业等5家企业在天津股权交易市场挂牌。五是深化医药卫生体制改革。按照国家和省的统一部署，落实好配套政策和资金，确保基本医疗保障、基本药物制度等五项重点工作落实到位。六是深化农村各项改革。建立健全征地补偿机制，稳定和完善农村基本经营制度，在依法自愿的基础上，促进土地适度规模经营和土地承包经营权流转。全面深化集体林权制度改革。探索农村房产确权改革和农民资产资本化的新途径。积极搞活农村金融，推进实施农业政策性保险，为农村发展注入活力。

（九）加快自主创新步伐，提高综合竞争力。围绕产业升级、企业升位和转变发展方式，加快引进、消化、吸收、再创新步伐，构建以企业为主体、市场为导向、产学研相结合的自主创新体系。一是着力壮大创新主体。进一步完善促进自主创新的政策措施，充分发挥政府科技创新专项资金的引导作用，推进河北华戈、新宝丰、华斯、东塑等企业研发中心和产品检验检测中心建设。加快智能化控制、PVC深加工、TDI工艺国产化等技术引进与研发，不断提高企业技术再创新能力。二是着力完善创新机制。加强与京津高等院校和科研单位的对接与合作，重点依托河北工业大学、北京化工大学等院所，联合建立重点实验室、工程技术中心、博士后工作站，根据我市产业发展需求实施重大创新专项攻关计划。打造创新平台，重点抓好沧州开发区国家级孵化器及市、县生产力促进中心建设。促进工业化与信息化有机融合，提高主导产业技术创新和应用水平。三是着力聚集创新人才。健全和完善有利于科技人才创新创业的评价、使用、激励机制，以重大科研项目、重点学科、关键技术为重点，引进科技创新人才，确保留得住、用得上。

（十）大力改善民生，积极构建和谐沧州。坚持把关注民生、重视民生、保障民生、改善民生作为基本职责，真心实意为群众办实事、办好事。一是全力做好就业再就业工作。坚持把促进就业作为经济发展的优先目标，着力做好困难家庭和大中专毕业生就业工作。年内全市新增就业4.5万人，城镇登记失业率控制在4.5%以内。二是进一步健全完善社会保障体系。加快以“六险三院一金全覆盖”为主的社会保障体系建设，进一步扩大“六险”覆盖面。积极做好青县、任丘、肃宁新型农民养老保险试点工作。加强“三院”管理，提高集中供养水平。加快新农合制度建设，提高新农合参合率和住院费用报销比例。三是着力提高住房保障能力。加大住房公积金归集力度，进一步扩大覆盖面，提高补贴标准。加快住房开发建设步伐，增加中小户型、中低价位普通商品房供给。推进廉租住房建设，新建廉租住房2500套。四是大力实施饮水安全工程。按照“引得来、蓄得住、用得好”的要求，制定饮水安全三年规划，实施水系水网工程，积极推进西部水库建设前期工作，加快杨埕水库向东部地区供水，大浪淀水库向孟村、盐山和泊头供水，力争用3年的时间，让全市人民告别苦咸水、高氟水，喝上黄河水、安全水。五是努力增加城乡居民收入。探索建立企业职工收入与经济发展同步增长机制，制定并落实好最低工资指导线标准。逐步提高企业退休人员基本养老金、部分优抚对象待遇和城乡居民最低生活保障水平。六是提升教育发展水平。进一步落实以县为主的义务教育经费保障机制，优化配置教育资源，促进义务教育均衡发展。大力改善办学条件，积极推进校舍安全工程。实施主城区教育专项规划和基础教育三年行动计划，加大中小学布局调整力度。整合教育资源，大力发展中、高等职业教育，建设与我市工业化进程相适应、以工科为主的综合类高等院校。七是积极做好卫生、人口和计划生育工作。努力提高县级医院的综合服务能力。强化医护人员培训，继续开展万名医师支援农村卫生服务活动。抓好基层医疗卫生服务网点建设，完善社区卫生服务功能，实现小病不出乡、大病不出县，为群众就医提供方便。全力做好甲型H1N1流感等重大传染性疾病防控工作。积极推动各项公共卫生事业的发展。完善利益导向机制，稳定低生育水平，综合治理出生人口性别比偏高问题。认真做好第六次人口普查工作。八是加快发展文化体育事业。完善基层公共文化体育设施，继续推进乡镇综合文化站和农村书屋建设，认真抓好110个国家级和232个省级农民健身工程。九是切实抓好安全生产和食品药品监管。严格落实安全生产责任制，以危化行业为重点，强化隐患排查和专项整治，防止重特大责任事故发生。探索建立食品安全监管长效机制，进一步开展食品及原油加工等生产领域专项整治。坚决打击非法传销犯罪活动，严厉查处取缔黑网吧，维护市场秩序。十是巩固社会和谐安定的基础。高度重视群众信访工作，积极开展民间矛盾纠纷排调，努力防范和化解各种社会矛盾，完善突发应急处置机制，有效预防和妥善处理各类群体事件。加强社会治安综合治理，依法打击各类违法犯罪活动，增强人民群众的安全感。

与此同时，支持工会、共青团、妇联等群众组织开展工作，发展妇幼、老龄、残疾人和红十字会等慈善事业。加强国防建设，做好“双拥”和民兵预备役工作。推动民族宗教、外事侨务、档案、气象、地震、测绘、人防等各项事业的发展。认真做好 “十二五”规划编制工作，力争成为体现省和国家战略意图、符合沧州实际的科学发展的好规划。

四、切实加强政府自身建设

打造冀中南区域发展的增长极和隆起带，推动沧州科学发展、率先发展、跨越发展，必须切实加强政府自身建设。

（一）加强理论武装，进一步提高科学发展的能力。坚持用科学发展观武装各级干部头脑，把科学发展观的要求贯穿政府工作的全过程，通过创新发展理念、转变发展方式、破解发展难题、提高发展质量，把科学发展观转化为推动发展的坚强意志、谋划发展的正确思路、领导发展的实际能力、促进发展的政策措施，构建保障科学发展的良好环境和体制机制。

（二）加强作风建设，进一步提高政府的战斗力。按照高标准、高效率、高水平的要求，深入开展干部作风建设年和“两转两提”活动，大力弘扬说干就干、干就干成、干就干好、干就一流的工作作风，以事不避难的昂扬锐气、拼搏进取的蓬勃朝气，努力创造沧州精神、沧州速度、沧州实力。

（三）加强制度建设，进一步提高政府的执行力。建立和完善各项政府规章制度，严肃工作纪律，坚决杜绝有令不行、有禁不止、各自为政、各行其是等不良行为，以责任明确的硬任务，真抓实干的硬措施，奖罚分明的硬机制，凝聚发展的强大合力，确保市委、市政府的各项决策部署落到实处。

（四）加强廉政建设，进一步提高政府的公信力。严格落实党风廉政建设责任制，健全完善惩防体系，确保行政权力规范运行、阳光操作。严格遵守廉洁自律的各项规定，切实加强党性锻炼和理想信念教育，保持高尚的精神追求，培养健康的生活情趣，做到不仁之事不做，不义之财不要，不正之风不沾，建设让党放心、让人民满意的政府。

（五）加强环境建设，进一步提高服务发展的能力。坚持一手抓硬环境的改善，继续加大基础设施建设力度，完善城市功能，提升城市品位，提高城市综合竞争能力。一手抓软环境的营造，着力改善投资环境、政策环境、人文环境，打造投资洼地、服务高地、创业福地。

（六）加强法制建设，进一步提高依法行政能力。严格实行行政执法责任制，规范行政行为，推进政务公开。自觉接受市人大及其常委会的法律监督、市政协的民主监督、新闻舆论和社会公众的监督，主动听取各民主党派、工商联、无党派人士及社会各界的意见和建议，认真办理人大代表建议和政协委员提案，自觉把政府工作纳入依法运行的轨道。

各位代表！沧州已进入快速发展的历史新阶段，时代赋予重托，奋斗铸就辉煌！让我们更加紧密地团结在以胡锦涛同志为总书记的党中央周围，在省委、省政府和市委的正确领导下，团结一心，抢抓机遇，顺势而为，乘势而上，为把沧州建设成为冀中南区域发展的增长极和隆起带而努力奋斗！
